# Effects of Improper Mechanical Force on the Production of Sonic Hedgehog, RANKL, and IL-6 in Human Periodontal Ligament Cells In Vitro

**DOI:** 10.3390/dj12040108

**Published:** 2024-04-15

**Authors:** Erika Yamashita, Shinichi Negishi, Jun Kikuta, Mami Shimizu, Hidenobu Senpuku

**Affiliations:** 1 Department of Orthodontics, Nihon University of School at Matsudo, Matsudo 271-8587, Japan; maer20024@g.nihon-u.ac.jp (E.Y.); negishi.shinichi@nihon-u.ac.jp (S.N.); kikuta.jun@nihon-u.ac.jp (J.K.);; 2 Department of Microbiology and Immunology, Nihon University of School at Matsudo, Matsudo 271-8587, Japan

**Keywords:** sonic hedgehog, RANKL, IL-6, hPDL

## Abstract

Improper mechanical stress may induce side effects during orthodontic treatment. If the roots and alveolar bones are extensively resorbed following excess mechanical stress, unplanned tooth mobility and inflammation can occur. Although multiple factors are believed to contribute to the development of side effects, the cause is still unknown. Sonic hedgehog (Shh), one of the hedgehog signals significantly associated with cell growth and cancer development, promotes osteoclast formation in the jawbone. Shh may be associated with root and bone resorptions during orthodontic treatment. In this study, we investigated the relationships between Shh, RANKL, and IL-6 in human periodontal ligament (hPDL) cells exposed to improper mechanical force. Weights were placed on hPDL cells and human gingival fibroblasts (HGFs) for an optimal orthodontic force group (1.0 g/cm^2^) and a heavy orthodontic force group (4.0 g/cm^2^). A group with no orthodontic force was used as a control group. Real-time PCR, SDS-PAGE, and Western blotting were performed to examine the effects of orthodontic forces on the expression of Shh, RANKL, and IL-6 at 2, 4, 6, 8, 12, and 24 h after the addition of pressure. The protein expression of Shh was not clearly induced by orthodontic forces of 1.0 and 4.0 g/cm^2^ compared with the control in HGFs and hPDL cells. In contrast, RANKL and IL-6 gene and protein expression was significantly induced by 1.0 and 4.0 g/cm^2^ in hPDL cells for forces lasting 6~24 h. However, neither protein was expressed in HGFs. RANKL and IL-6 expressions in response to orthodontic forces and in the control were clearly inhibited by Shh inhibitor RU-SKI 43. Shh did not directly link to RANKL and IL-6 for root and bone resorptions by orthodontic force but was associated with cell activities to be finally guided by the production of cytokines in hPDL cells.

## 1. Introduction

The human periodontal ligament (hPDL) and the alveolar bone are parts of the periodontium, which is a complicated structure that supports a tooth. The alveolar bone and periodontal tissues formed in conjunction with a tooth are linked to the root surface by collagen fibers of the PDL [1,2]. Fibroblasts are the main cell type of the PDL, which additionally includes epithelial cells and undifferentiated mesenchymal stem cells [3]. PDL cells have been extensively studied in vitro for their differentiation into osteoblast-like cells and their supportive role in osteoclastogenesis. These cells can contribute to both osteogenesis and osteoclastogenesis [4]. Human PDL cells have the characteristic phenotype of osteoblast-like cells [5] and can initiate mineral-like nodules in vitro [6]. PDL fibroblasts (PDLFs) can attract osteoclast and odontoblast precursor cells, and these cells move to the bone and root surfaces and can cause bone and root resorption [7,8,9]. 

Orthodontic tooth movement (OTM) by orthodontic forces is based on the principle that a tooth moves into a bone. Excess mechanical stress may induce side effects during orthodontic treatment. If the roots and alveolar bones are extensively resorbed following excess mechanical stress, unplanned tooth mobility and inflammation can occur. Although multiple factors are thought to contribute to the development of side effects, the cause is still unknown. 

The palmitoylation of the hedgehog (Hh) family of morphogens, named sonic hedgehog (Shh), desert hedgehog (Dhh), and Indian hedgehog (Ihh), is important for effective short-range and long-distance signal transmission. Hedgehog acyltransferase (Hhat) links palmitic acid molecules to Hh. Therefore, the variation in Hhat causes the expansion of the phenotype. Hh are members of the secretion signal transmission protein family mediating growth and pattern formation during embryo generation [10,11]. These proteins function as morphogens and form a signal transmission incline for long-distance and short-range interaction [12]. This phenomenon is developed by differentiation of intestinal epithelium cells, and Ihh and Shh send a signal to a patched (PTCH) receptor. The active form for the Hh ligand reduces the inhibition effect on the G-protein-coupled receptor Smoothened (SMO) when interacting with PTCH and activates the zinc finger glioma-associated oncogene transcription factor (GLI). The Hh signal transmission derives the maturity of the osteoclast indirectly and promotes bone resorption [13]. In another in vitro study, Shh upregulated the Osx expression in the osteoblastic stock [14] and increased the production of osteoblasts, and it raised the activity of the osteoclast indirectly. As a result, bone resorption increased, and it was shown that bone strength decreased [13,14,15]. The extreme expression of Hh and the Wnt/β- catenin signal transmission causes either ectopic ossification in insufficient osteoplasty or soft tissue in frame-like osteoporosis.

The osteoblasts promote differentiation of the osteoclast by producing receptor activator nuclear factor κB (RANK) and interacting nuclear factor κB receptor activation factor ligand (RANKL) on the surface of the osteoclast progenitor cell [16,17]. However, these cells express osteoprotegerin (OPG), which is the decoy receptor of the RANKL. OPG inhibits activation of NF-κB, which is a central and fast-acting transcription factor for differentiation to the osteoclast in immune stimulation, and the precursor cell by binding to RANKL [18]. It has been reported that cytokines, IL-6 in particular, affect the RANK-RANKL-OPG system to promote differentiation of the osteoclast [16]. 

Mammalian Hh with the broadest expression pattern including developing nervous system, limbs bud, skin, and the bowels has been studied in Shh. The Shh pathway plays an important role in activation with differentiation of the osteoclast [19]. The Ihh signal pathway is a development and an important adjustment factor of homeostasis [20], and it is necessary to strengthen Ihh signal transmission to promote bone formation [21]. The expression of Dhh is mainly found in the gonads and testes, and some expression is found in peripheral nerves and the pancreas. Among Hhs, Shh may be associated with root and bone resorptions in the development of side effects during orthodontic treatment. However, the relationships Shh has with the production of IL-6 and RANKL, which are involved in root resorption and bone resorption, have not been clearly demonstrated in previous studies. In this study, we investigated the stress responses of PDLF cells and the relationships between Shh, RANKL, and IL-6 in PDLFs exposed to weights corresponding to optimal and heavy orthodontic forces in vitro. As a control, human periodontal gingival fibroblast (HGF), which does not play a role in root resorption, was also used for the research of responses to mechanical stresses. The relationships were indicated by changes in cell morphology, cell activity, and expression of the RANKL and IL-6 genes and proteins. These results may be helpful for clarification of the mechanisms involved in the development of side effects caused by mechanical stress during orthodontic treatment. Therefore, it may be possible to identify targets for the prevention of the side effects of orthodontic treatment.

## 2. Materials and Methods

### 2.1. Cell Culture and Application of Compression Force

We purchased cells of human PDLFs (Number: 2630, Lot: 22328, ScienCell Research Laboratory, Carlsbad, CA, USA) and human periodontal gingival fibroblasts (HGFs, KF-4309, Kurabo, Osaka, Japan), as typical cells, which are isolated from human periodontal tissue. A cells were used as an another PDLF. The A cells were collected from the roots of premolars extracted from one young volunteer (21 years old) during course of orthodontic treatment after obtaining informed consent from the donor, and the PDLFs were used according to a protocol examined by the Ethical Review Board of Nihon University School of Dentistry at Matsudo (EC 20-036). Human gingival epithelial Ca9-22 cells were also obtained from the JCRB cell bank (RIKEN, Saitama, Japan) and used as a second control. Second- to fifth-passage human PDL cells were seeded in duplicate into a Petri dish (100-mm) and cultured to confluence prior to further experimental stimulation in α-MEM containing 10% fetal bovine serum and 1% antibiotics (diluted from a stock solution containing 10,000 U/mL of penicillin and 10,000 U/mL of streptomycin; Biochrom AG, Berlin, Germany) at 37 °C in an atmosphere of 100% humidity, 95% air, and 5% CO_2_ [22]. The culture medium was changed to 1% FBS α-MEM with penicillin and streptomycin, and a cover glass and weights were placed—for the optimal orthodontic force group (1.0 g/cm^2^) and the heavy orthodontic force group (4.0 g/cm^2^)—on the top of the PDLF and HGF cell layers for 2, 4, 6, 8, 12, and 24 h cultures [23,24]. A cover glass (0.052 g/cm^2^) was only placed for the control of the weight, and a combination of no glass and no weight was also used as a negative control. For clarification of the role played by Shh in the effect of weight on the production of RANKL and IL-6, 10 mM of Shh inhibitor (RU-SKI 43, Med Chem Express, Monmouth Junction, NJ, USA) was added in the culture of PDLFs with weight and the controls [25]. RU-SKI 43 is a potent and selective hedgehog acyltransferase (Hhat) inhibitor with an IC50 of 850 nM. RU-SKIs inhibit Shh palmitoylation in vitro [25]. RU-SKI 43 decreases proliferation and the Gli-1 activation of the cancer cells of the pancreas through noncanonical signal transmission not dependent on Smoothened [26]. Therefore, RU-SKI 43 was selected among the inhibitors because RU-SKI 43 is an inhibitor of the sonic hedgehog signal and inhibits the first signal of sonic hedgehog. After 24 h, 2.0 × 10^6^ of CA9-22 cells, 1.6 × 10^6^ of hPDLF cells, and 1 × 10^6^ of A cells and HGF cells were propagated on the dish.

### 2.2. Measurement of Lactate Dehydrogenase (LDH)

LDH is widely used both in vivo and in vitro as a marker of cell death. The release of LDH to a tissue culture nutrient medium exactly reflects in vitro cell survival rate. We investigated the relationship between cell density and total LDH activity in cell culture supernatant samples. LDH was measured by the Cytotoxicity LDH Assay kit-WST (Dojindo Molecular Techbologies, Inc., Kumamoto, Japan).

### 2.3. Measurement of Cell Viability by MTT

Four ml of MTT (Sigma Chemical Co., Dorset, UK), 500 μg/mL in PBS were added to HGF, PDLF, A cells, and Ca9-22 cells in each Petri dish at 24 h after incubation. The cells were incubated for 3–4 h at 37 °C, under CO_2_. After 3–4 h, these cells turned a purple color from the formal form of the cells. MTT was discarded, two mL of dimethyl sulfoxide (Wako Pure Chemical Industries Ltd., Osaka, Japan) was added to each Petri dish. The plate was wrapped using aluminum foil, left for 24 h at room temperature, and then re-absorption of 492 nm was performed using a microplate absorbance meter (Corona Electronic, Ibaraki, Japan).

### 2.4. SDS-PAGE and Western Blotting

At 6 h after culture start, culture supernatants were extracted as a 0~6 h sample and fresh 10%FBS α-MEM were added, and the culture was continued. At 18 h after culture re-start, culture supernatants were extracted as a 6~24 h sample. Therefore, the 0~6 h sample included products from cell stimulation for 0~6 h. The 6~24 h sample included products from cell stimulation for 6~24 h. Before electrophoretic analysis, these culture supernatants were concentrated by a centrifugal filter (Amicon^®^Ultra-15, 50 kDa, Merk Millipore Ltd., Burlington, MA, USA) with more than 50 kDa and diluted to 100 μg in 10 μL with an equal volume of SDS-PAGE buffer {0.06 M Tris-HCl (Amersham Pharmacia Biotech, Buckinghamshire, UK), pH 6.8 of the UK, 20% glycerol (Wako), 1% (*w*/*v*) SDS (Wako), 1% 2-mercaptoethanol (2-ME, Merck kGaA, Darmstadt, Germany), and 0.0012% of bromophenol blue (Wako)}. The SDS-PAGE sample was heated to 100 °C for 5 min before it was loaded to a gel. The samples were then separated into 12.5% polyacrylamide gel (e-PAGEL, ATTO Corp., Tokyo, Japan) in 0.025 M Tris-HCl, 192 mM glycine (Wako) and 0 or 0.1% (*w*/*v*) SDS. The separation by electrophoresis of the protein was carried out at 25 mA for 80 min. The staining of the gels was performed with Coomassie Brilliant Blue (CBB, Wako).

After SDS-PAGE, the proteins in the gel were transferred to Immobilon PVDF membranes (Millipore, Bedford, MA, USA), and the membrane were blocked with 2.0% skimmed milk in TBST (50 mM Tris, 2.7 mM KCl, 0.138 M NaCl, 0.05% Tween 20, pH 7.6) for 1 h at room temperature. The membranes were applied with anti-Shh antisera (Bioss Antibodies, Woburn, MA, USA), anti-RANKL antisera (Bioss Antibodies), and anti-IL-6 antisera (Protein Tech Group Inc., Rosemont, IL, USA) from rabbit in 1.0% skimmed milk/TBST overnight at 4 °C. After washing with TBST, HRP-labeled anti-goat secondary antibodies to rabbit IgG (Merck kGaA) were also applied to react with the target proteins. Optical emission responses to the target proteins were identified by chemiluminescence (ECL Plus Western Blotting Substrate, Thermo Scientific, Southfield, MI, USA) and detected by exposure to X-ray film (FUJI FILM, Kanagawa, Japan) for different times. Protein bands were quantitatively measured by ImageJ, 1.53t (NIH, MD, USA). The measurement range of brightness was set according to the area of each Shh, RANKL, or IL-6 band, and various controls, weights, and time conditions were compared and examined.

### 2.5. Quantitative Measurement of RNAKL and IL-6

The productions of RNAKL and IL-6 in culture supernatants were detected by ELISA kit (RANKL: Abcam, Cambridge, UK and IL-6; Wako).

### 2.6. Total RNA Extraction and Real-Time PCR

PDLF cells were collected following force loading. After incubation for various times, total RNA was taken by an RNeasy Mini Kit (Qiagen, Hilden, Germany). Next, 0.5 µg of total RNA was reverse-transcribed using the Prime Script^TM^ RT Reagent Kit (TaKaRa BioInc., Shiga, Japan) to obtain complementary DNA (cDNA).

Quantitative reverse transcription PCR (RT-qPCR) was performed using an ABI QuantStudio 7 Flex (Applied Biosystems, Warrington, UK) using the Power SYBR Green PCR master mix (Applied Biosystems), as previously described [27]. The expression levels of the genes were measured by the relative quantitative comparative threshold cycling method (ΔΔCT). The two kinds of cycling conditions were as follows: initial denaturation cycling conditions of 10 min at 95 °C, 40 cycles of 15 s at 95 °C, and 60 s at 60 °C for the gene of Shh; and 5 min at 95 °C, 45 cycles of 10 s at 95 °C, and 20 s at 60 °C for the genes of RANKL and IL-6. The expression levels were normalized to the GAPDH mRNA (endogenous control). The primers used for qPCR are listed in Table 1.

### 2.7. Statistical Analysis

Statistical analyses were performed using Statistical Package for Social Sciences (SPSS) statistics 24 (IBM Corporation, Armonk, NY, USA). The results are presented as the mean and standard deviation (SD). The statistical significance of differences between the negative control (no glass and no weigh), the control (glass only), and the 1.0 g/cm^2^ and 4.0 g/cm^2^ groups was determined in 2~24 h using one-way analysis of variance (ANOVA) and Bonferroni (IBM SPSS statistics 24) tests. The statistical significance was set at *p* < 0.05.

## 3. Results

### 3.1. Effects of Orthodontic Force on the Cell Activities, Adherence, and Morphology

**Figure 1:**
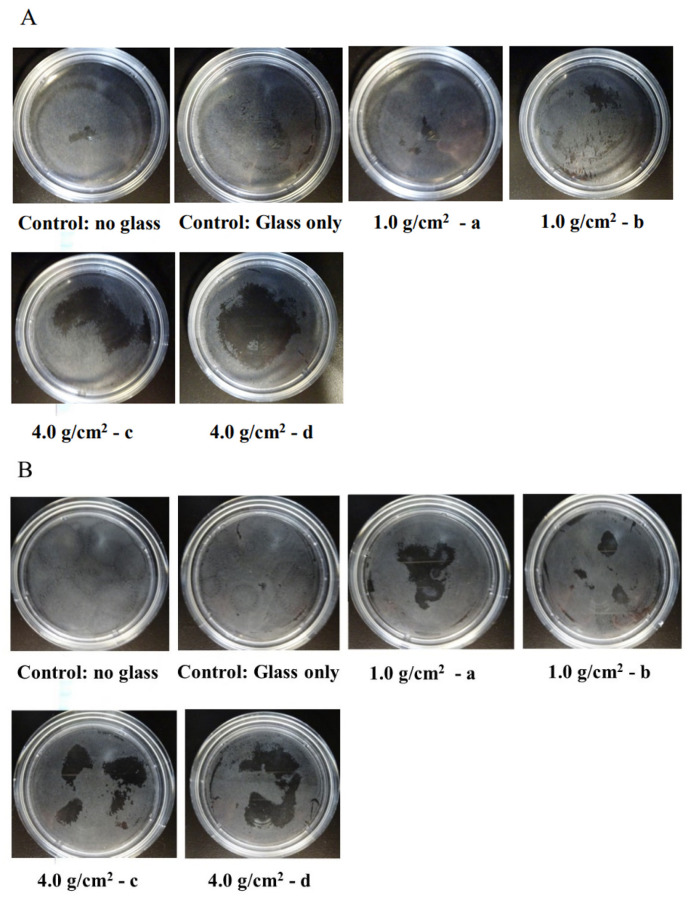
Effects of compression force on the fibrosed layers of HGF and the PDLF cells. The optimal orthodontic force (1.0 g/cm^2^, a and b) and the strong orthodontic force (4.0 g/cm^2^, c and d) were, in duplicate, placed on HGF (**A**) and PDLF (**B**) confluent cells in Petri dishes. Glass was only used as a control. No glass and no weights was also used as a negative control. After 24 h, the HGF and PDLF cells were photographed and observed using a camera. The white area shows the cells that remained and the black area shows the PDLF cells that were removed from the Petri dish. Representative data from more than three independent experiments are presented in the form of pictures.

**Figure 2:**
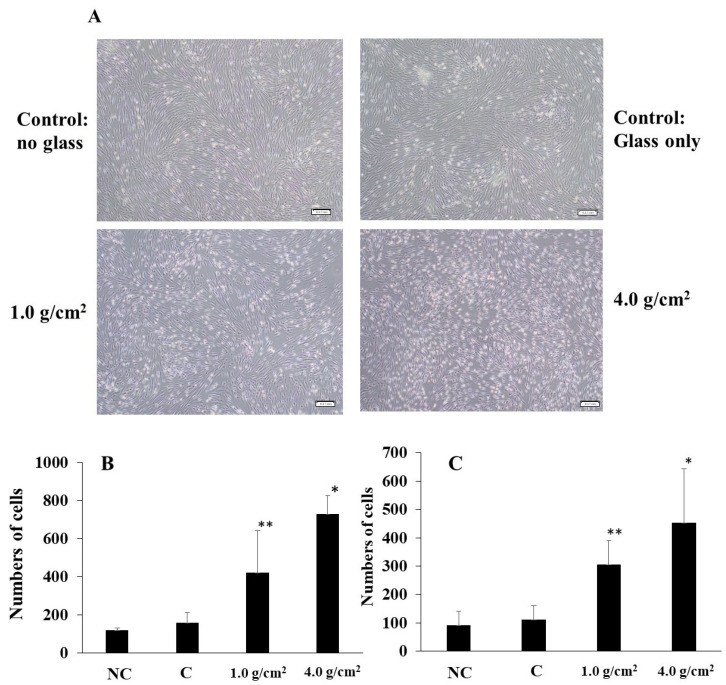
Observation of white round cells under a compression force on the fibrosed layers of PDLF cells. An optimal orthodontic force (1.0 g/cm^2^) and a strong orthodontic force (4.0 g/cm^2^) were placed on PDLF-confluent cells in Petri dish. No glass and no weights was also used as a negative control (NC). Glass was only placed as a control (C). After 24 h, the PDLF cells were observed and pictures were taken using a microscope (**A**). Representative data from more than three independent experiments are presented in the pictures. White round cells in the HGF (**B**) and PDLF (**C**) cells were counted in 2.16 × 1.6 mm^2^ pictures. The scale bar indicates 0.17 mm. At least five pictures were taken in each experiment. The data indicate the mean ± SD of three independent experiments. The asterisks indicate a significant difference at 24 h after the culture in the NC, C, 1.0 g/cm^2^ group, and 4.0 g/cm^2^ group (NC, C, and 1.0 g/cm^2^ vs. 4.0 g/cm^2^ group, *: *p* < 0.05, NC and C vs. 1.0 g/cm^2^, **: *p* < 0.05).

**Figure 3:**
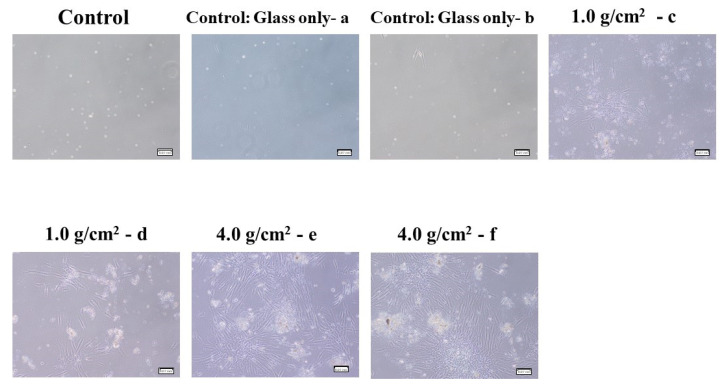
Observation of PDLF cells removed by compression force on the fibrosed layers of PDLF cells. The optimal orthodontic force (1.0 g/cm^2^, c and d), the strong orthodontic force (4.0 g/cm^2^, e and f), and control (glass only (a and b)) were placed on PDLF-confluent cells in a Petri dish. No glass and no weights was also used as a negative control. After 24 h, the PDLF cells were detached when removing the glass and washing with fresh medium. The detached cells were re-incubated on the Petri dish in fresh 10%FBS α-MEM. Some of the grown cells were attached and observed by microscope. The scale bar indicates 0.07 mm. Representative data from more than three independent experiments are presented in the pictures.

**Figure 4:**
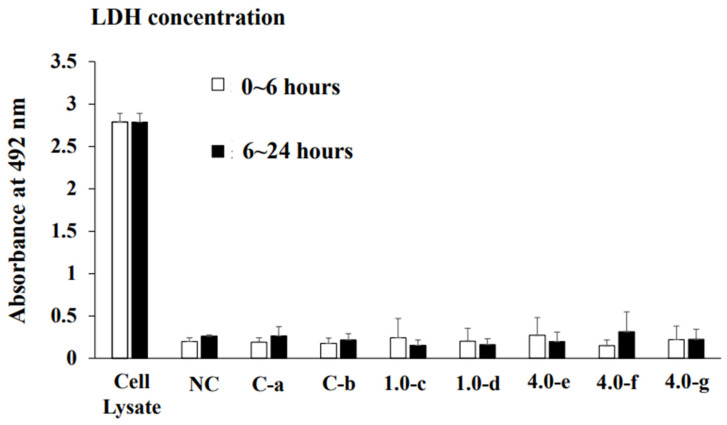
Quantitative analysis of LDH in the culture supernatant of PDLF cells with 1.0 g/cm^2^ and 4.0 g/cm^2^ weights. The optimal orthodontic force (1.0 g/cm^2^, c and d) and the strong orthodontic force (4.0 g/cm^2^, e, f and g) were placed on PDLF-confluent cells in Petri dishes. Glass only was used as a control (a and b). No glass and no weight was also used as a negative control. At 6 h after the start of the culture, culture supernatants were extracted as a 0~6 h sample and fresh 10%FBS α-MEM was added and the culture was continued. At 18 h after culture re-start, culture supernatants were extracted as a 6~24 h sample. The cells were lysed by lysis buffer and the lysed cells were used as a positive control. LDH was measured in all samples. The data indicate the mean ± SD of triplicate experiments. The independent experiments were performed three times, with similar results obtained in each experiment.

**Figure 5:**
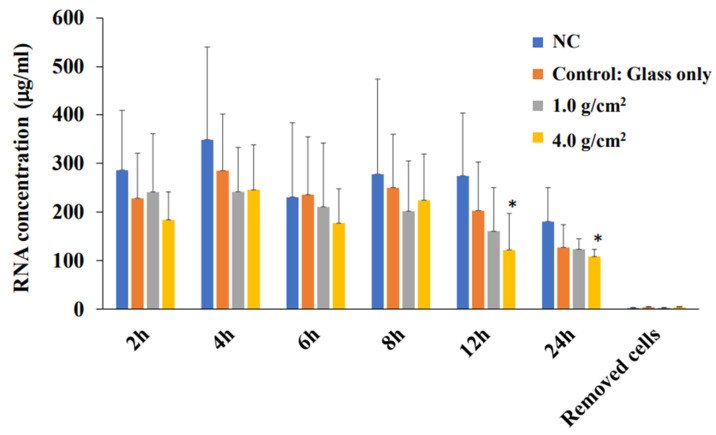
RNA concentration at various times after culture in PDLF cells with weights. The optimal orthodontic force (1.0 g/cm^2^) and the strong orthodontic force (4.0 g/cm^2^) were placed on PDLF-confluent cells in Petri dishes. Glass only was used as a control. No glass and no weights was also used as a negative control (NC). At 2, 4, 6, 8, 12, and 24 h after culture start, total RNA was extracted from the attached PDLF cells and the removed cells at 24 h. The data indicate the mean ± SD of three independent experiments. The asterisks indicate a significant difference between RNA concentrations at various times in the 4.0 g/cm^2^ group (one way ANOVA analysis, *: *p* < 0.05).

**Figure 6:**
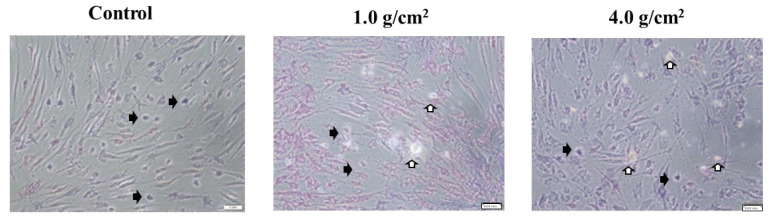
Observation of white round cells in PDLF cells stained by MTT. Cell viabilities were observed using the MTT method. The optimal orthodontic force (1.0 g/cm^2^) and the strong orthodontic force (4.0 g/cm^2^) were placed on various cells in Petri dishes. The glass plate only was only used as a control. After 24 h, the cells were observed and pictures were taken using a microscope (×200 magnification). White round cells stained (black arrow) and not stained (white arrow) by MTT were observed in PDLF cells under 1.0 g/cm^2^ and 4.0 g/cm^2^ weights. The scale bar indicates 0.04 mm. At least five pictures were taken in each experiment. Representative data from two independent experiments were presented in the pictures.

To clarify the effects of compressive force on the expression of Shh, RANKL, and IL-6 in HGF and PDLF, an optimal orthodontic force (1.0 g/cm^2^) and a strong orthodontic force (4.0 g/cm^2^) were placed on the fibrosed layers of HGF and the PDLF cells and incubated for 2, 4, 6, 8, 12, and 24 h. The heavy force may repress cell viability and increase the mortality rate of hPDL [24]. First, we observed whether these compression forces damage the cells. HGFs were slightly and strongly detached by 1.0 g/cm^2^ and 4.0 g/cm^2^ weights after 24 h of culture, respectively (Figure 1A). In particular, cells were widely removed from the surface of the Petri dish and principally around the center at 24 h. The PDLF cells were largely detached by 1.0 g/cm^2^ and 4.0 g/cm^2^ weights in various places and the areas of the detached cells were fused at 24 h of culture (Figure 1B). The levels of the detached areas were larger in the 4.0 g/cm^2^ weight group than in the 1.0 g/cm^2^ group. There were no detached cells at 2–12 h in all conditions after culture in either cell line. Therefore, the weight and the pressure time were associated with the removal of the HGF and the PDLF cell fibrosed layers on the surface of the Petri dishes. However, both cell types were not detached by controls (glass only weight and no glass and no weight) (Figure 1A,B). Next, to confirm whether cell morphology was associated with the detachment of cells by weights, we observed PDLFs using an optical microscope after 24 h culture. The cells attached to the Petri dish in the form of fibers, but we confirmed that the number of white round cells appeared to be dependent on the weight (Figure 2A–C). The number of white round cells was significantly higher when a 4.0 g/cm^2^ weight was placed on the cells than when a 1.0 g/cm^2^ weight was placed on the cells. The number of white round cells increased, and this change might be the trigger for the detachment of the cells (Supplemental Figures S1 and S2). Therefore, the detachment of the cells is not only due to the physical force when removing the glass but also because the cell shape changes from fibrous to spherical due to the weight. The change in cell morphology may be associated with cell damage induced by the pressure. To confirm whether the detached cells are living or dead cells, we recultured the detached cells in fresh 10%FBS α-MEM on a Petri dish. The cells removed from the 1.0 g/cm^2^ and 4.0 g/cm^2^ groups were largely reattached, increased, and showed a fibrous form on the Petri dish (Figure 3). This finding indicates that the detached cells were not dead. LDH, which is released when the cell membrane ruptures and the cells die, was measured in the culture supernatants from PDLF cultures placed with the controls and the 1.0 g/cm^2^ and 4.0 g/cm^2^ groups. LDH was not significantly produced in any of the cultures (Figure 4). Total RNA, as an indicator of the activity of the cells, was measured in the cells. However, total RNA was not found in the cells detached from the PDLFs with 1.0 g/cm^2^ and 4.0 g/cm^2^ weights (Figure 5). Therefore, the removed cells are in suspended animation and can be reverted to live cells when cultured in fresh medium. To measure cell viability in conditions with weights, an MTT assay was performed in gingival fibroblast (HGF), periodontal ligament fibroblasts (PDLFs and A cells), and epithelial cells (Ca9-22) at 24 h. Viability was significantly decreased by cover glass only (C) compared with NC in all cells, and decreased further still by placement of a 1.0 g/cm^2^ weight on the cover glass in Ca9-22 and by placement of a 4.0 g/cm^2^ weight in HGF, PDLF, A cells, and Ca9-22 cells (Supplemental Figure S3). Cell activity was significantly decreased by mechanical stress, including by a light weight such as the glass plate. The decreased levels compared with C were higher for the 4.0 g/cm^2^ weight than the 1.0 g/cm^2^ weight, and between C and the 4.0 g/cm^2^ weight were higher in HGF and Ca9-22 than PDLF and A cells. Cells removed after 24 h of culture with the 4.0 g/cm^2^ weight were slightly stained by MTT in Ca9-22 and HGF but not in PDLF and A cells. Therefore, the live cells were slightly removed by the weights in Ca9-22 and HGF. In contrast, the active cells remained after 24 h in PDLF and A cells with the 4.0 g/cm^2^ weight. To confirm the viabilities of white round cells stained by MTT in PDLF cells compressed by weights, the observation was performed using a strong magnification (×200) for the microscope. White round cells both stained and not stained by MTT were observed in the PDLF cells with the 1.0 g/cm^2^ and 4.0 g/cm^2^ weights but, white round cells that were not stained with MTT were not observed in the control (Figure 6).

### 3.2. Effects of Orthodontic Force on the Expression of Shh, RANKL, and IL-6 from PDLF Cells

**Figure 7:**
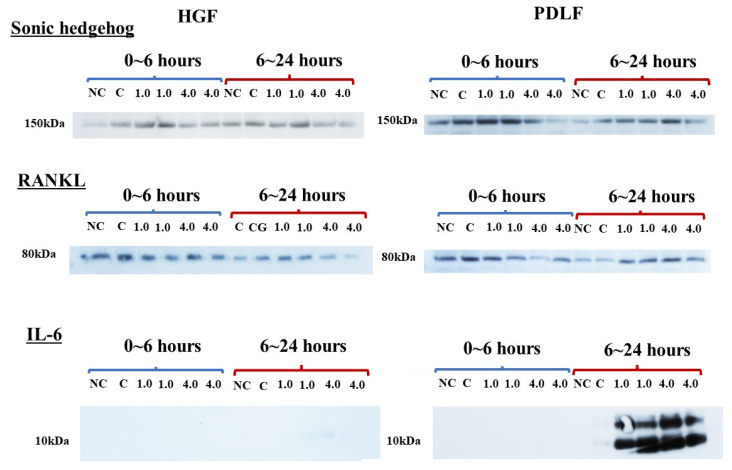
Observation of protein bands for Shh, RANKL, and IL-6 in culture supernatants of the HGF and the PDLF cells under different weights. The optimal orthodontic force (1.0 g/cm^2^) and the strong orthodontic force (4.0 g/cm^2^) were, in duplicate, placed on HGF and PDLF-confluent cells in Petri dishes. Glass only was used as a control (C). No glass and no weights was also used as a negative control (NC). At 6 h after culture start, culture supernatants were extracted as a 0~6 h sample and fresh 10%FBS α-MEM was added and the culture was continued. At 18 h after culture re-start, culture supernatants were extracted as a 6~24 h sample. SDS-PAGE and Western blotting using antibodies for Shh, RNAKL, and IL-6 were performed for the 0~6 h and 6~24 h samples, and positive bands were presented and compared each other. Representative data from more than three independent experiments are presented in the pictures.

**Figure 8:**
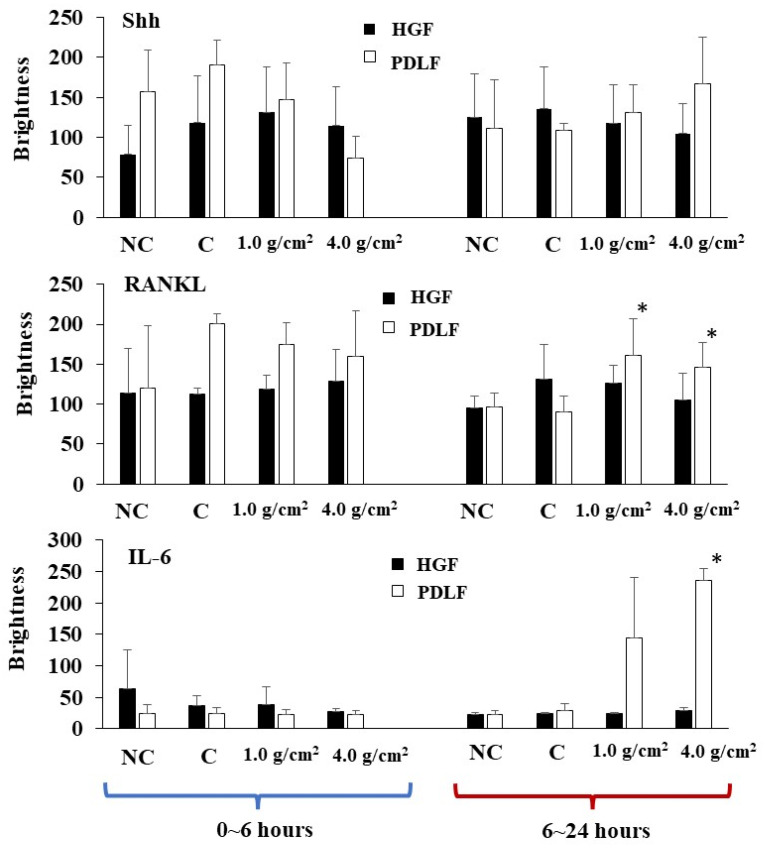
Quantitative analysis of protein bands for Shh, RANKL, and IL-6 in culture supernatants of the HGF (black bar) and the PDLF (white bar) cells under different weights. Protein bands in Figure 7 were quantitatively measured by ImageJ. Glass only was used as a control (C). No glass and no weights was also used as a negative control (NC). The results of brightness were expressed as the means ± SD of three independent experiments. The asterisks indicate a significant difference in brightness of the 1.0 g/cm^2^ group or the 4.0 g/cm^2^ group (vs. NC and C, *: *p* < 0.05).

**Figure 9:**
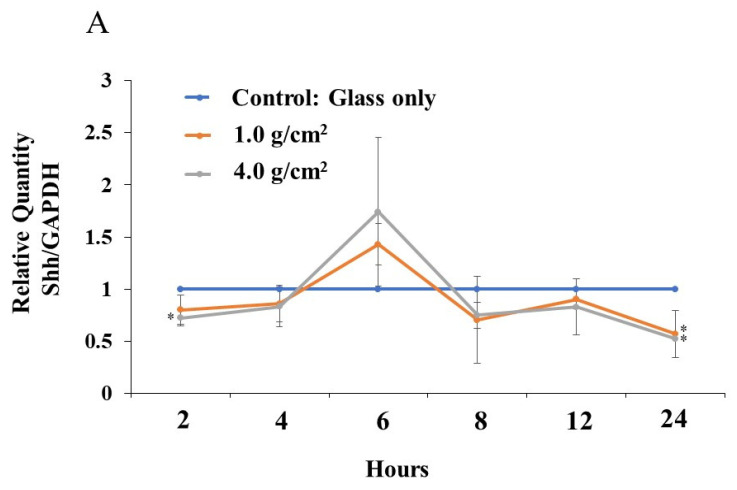

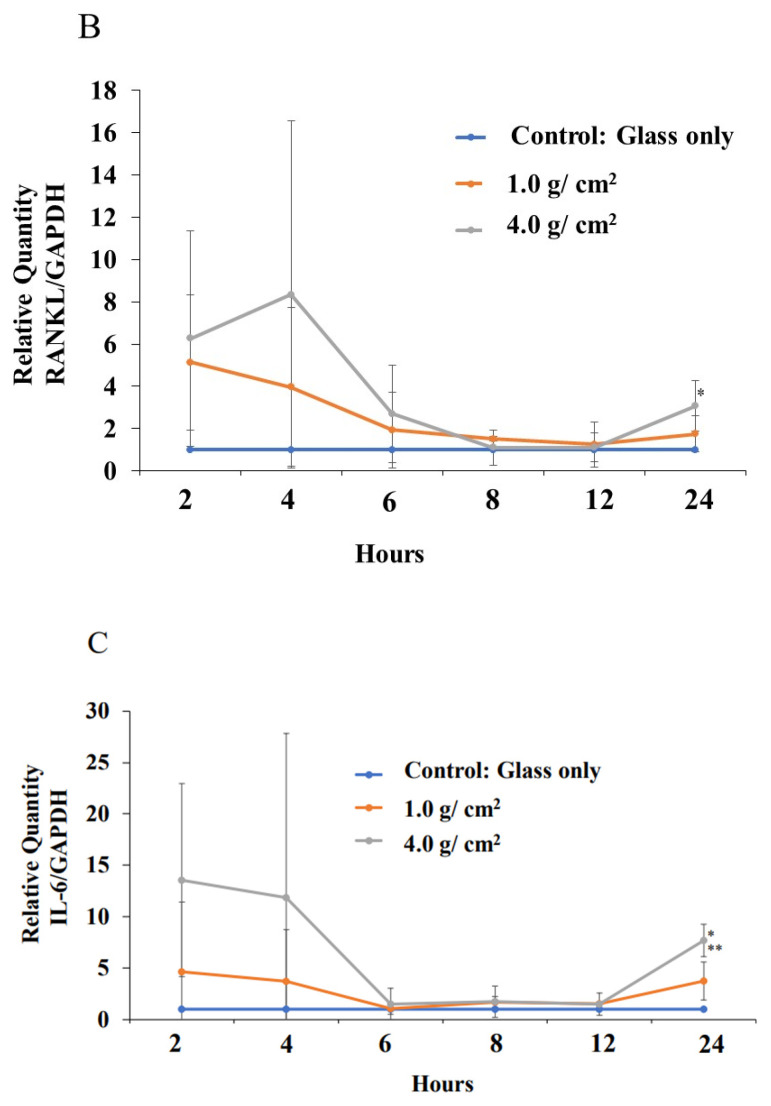
The gene expression of Shh, RANKL, and IL-6 at various times after culture in PDLF cells under different weights. The optimal orthodontic force (1.0 g/cm^2^) and the strong orthodontic force (4.0 g/cm^2^) were placed on PDLF-confluent cells in Petri dishes. Glass only was used as a control. After extraction of total RNA, real-time PCR was performed to detect the gene expression of Shh (**A**), RANKL (**B**), and IL-6 (**C**). The results are expressed as the means ± SD of three independent experiments. The asterisks indicate a significant difference among RNA concentration at various times (control vs. 1.0 g/cm^2^ or 4.0 g/cm^2^ group, *: *p* < 0.05, 1.0 g/cm^2^ vs. 4.0 g/cm^2^ group, **: *p* < 0.05).

In the HGF and PDLF cell cultures with 1.0 g/cm^2^ and 4.0 g/cm^2^ weights, we examined how Shh, RANKL, and IL-6 are produced by weight stimulation. To measure the protein production of Shh, RANKL, and IL-6, we performed Western blotting of the culture supernatant samples from the PDLF culture with no weight, control (glass only weight) and the 1.0 g/cm^2^ and 4.0 g/cm^2^ groups. The culture supernatant samples were concentrated to 50 kDa by filtration and prepared for SDS-PAGE and Western blotting. Moreover, the samples below 50 kDa that passed through the filter were used to compare to the more than 50 kDa sample in experiments. A protein band of around 150 kDa was mainly detected by anti-hedgehog antibodies in the 1.0 g/cm^2^ weight group and was similar to that in the control (glass only) group in both cells (Figure 7). In contrast, the 150 kDa protein band was poorly expressed in the 4.0 g/cm^2^ group, except for one protein band of PDLFs at 6~24 h. In RANKL, approximately 80 kDa protein bands were mainly detected and protein expression levels were not higher in the 1.0 and 4.0 g/cm^2^ weight groups than in the control (glass only) weight group at 0~6 and 6~24 h after culture in HGF and at 0~6 h in PDLFs (Figure 7). The expression of RANKL proteins was higher in the 1.0 g/cm^2^ and 4.0 g/cm^2^ groups than in the control (glass only) weight group at 6~24 h after culture in PDLFs. For IL-6, approximately 10 kDa protein bands were mainly detected, and protein expression levels were significantly higher in the 1.0 and 4.0 g/cm^2^ weight groups than in the control (glass only) weight group at 6~24 h after culture in PDLF cells (Figure 7). There were no positive bands in HGF and PDLF cells at 0~6 h, and in HGF at 6~24 h after culture. Positive bands were not detected in the less than 50 kDa samples. To measure the proteins quantitatively, the expression levels of these protein bands in Figure 7 were analyzed by ImageJ. In HGF, there were no significant differences in any proteins in the various conditions (Figure 8). In contrast, the protein bands of RANKLs were significantly expressed in the 1.0 g/cm^2^ and 4.0 g/cm^2^ groups in the 6~24 h after culture samples. The protein band of IL-6 was significantly expressed in the 1.0 g/cm^2^ group at 6~24 h after culture. If the protein is expressed, the gene must also have previously been expressed. To measure the gene expression of Shh, RANKL, and IL-6 in PDLFs, we collected total RNA samples, and their concentrations were first measured in all conditions. The amounts of total RNA significantly decreased in a time-dependent manner, and lower levels were significantly detected at 12 and 24 h at the 4.0 g/cm^2^ weight (Figure 5). The amounts of RNA also decreased in a time-dependent manner at the 1.0 g/cm^2^ weight, but the decease was not significant. The amounts of RNA were not largely changed at 2~12 h and decreased at 24 h, whereas there were no significant changes in the controls. Next, the gene expression levels of Shh, RANKL, and IL-6 were quantitatively measured and compared between the 1.0 g/cm^2^ and 4.0 g/cm^2^ weight groups and the control (glass only) weight group. Data are shown as relative quantity (RQ) to GAPDH of the control (glass only) weight group. The gene expression of Shh was significantly lower at 2 h after incubation in the 4.0 g/cm^2^ group, increased and was significantly higher at 6 h, and then decreased at 8 h and was significantly lower in the 1.0 g/cm^2^ and 4.0 g/cm^2^ weight groups than in the control (glass only) weight group at 24 h after incubation (Figure 9A). The gene expression of RANKL was higher at 2 and 4 h with no significant differences, decreased at 6 h, and was significantly higher at 24 h after incubation with the 4.0 g/cm^2^ weights than with the control (glass only) group (Figure 9B). There was no significant difference in gene expression of RANKL between the 1.0 g/cm^2^ weight and the control at 24 h after incubation. The gene expression of IL-6 was higher at 2 and 4 h with no significant differences, decreased at 6 h, and was significantly higher at 24 h after incubation with the 4.0 g/cm^2^ weight than with the 1.0 g/cm^2^ weight and the control (glass only) weight (Figure 9C). There were no significant differences in the gene expression of IL-6 between the 1.0 g/cm^2^ weight and the control.

### 3.3. Effects of Shh on the Expression of RANKL and IL-6 from PDLF Cells

**Figure 10:**
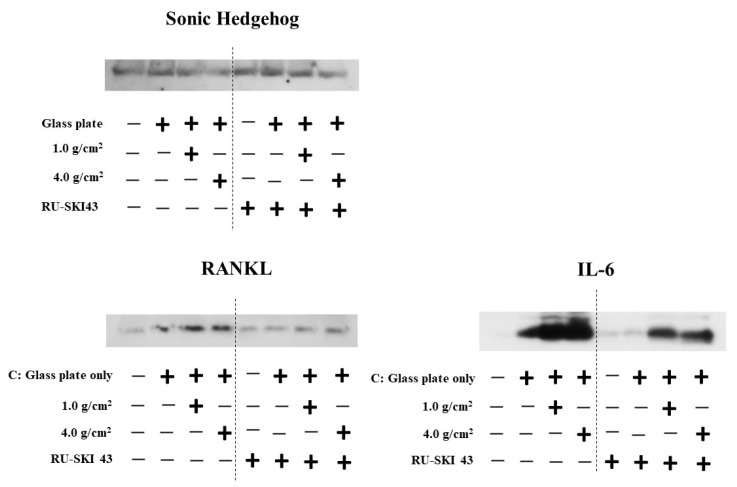
Observation of the relationship between Shh and RANKL or IL-6 in culture supernatants of the PDLF cells under different weights. The optimal orthodontic force (1.0 g/cm^2^) and the strong orthodontic force (4.0 g/cm^2^) were placed on PDLF-confluent cells. The cultures were performed with and without RU-SKI 43, an Shh signal inhibitor. Glass only was used as a control. At 6~24 h after culture, culture supernatants were extracted, and SDS-PAGE and Western blotting using antibodies to Shh, RNAKL, and IL-6 were performed in the 6~24 h samples. Positive bands were presented and compared to each other. Representative data from more than three independent experiments are presented in the pictures.

**Figure 11:**
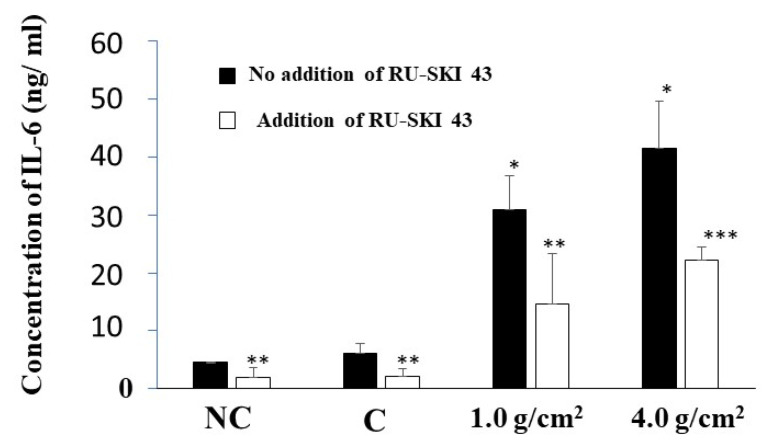
Quantitative measurement of IL-6 in culture supernatants of the PDLF cells under different weights. The optimal orthodontic force (1.0 g/cm^2^) and the strong orthodontic force (4.0 g/cm^2^) were placed on PDLF-confluent cells. The culture samples with and without RU-SKI 43, an Shh signal inhibitor, in Figure 10 were quantitatively measured by ELISA. Glass only was used as a control (C). No glass and no weights was also used as a negative control (NC). The results of IL-6 concentration were expressed as the means ± SD of three independent experiments. The asterisks indicate a significant difference in 1.0 g/cm^2^ group or 4.0 g/cm^2^ group (vs NC and C, *: *p* < 0.05, **: *p* < 0.05, addition of RU-SKI 43 vs. no addition of RU-SKI 43 and ***: *p* < 0.01, addition of RU-SKI 43 vs. no addition of RU-SKI 43).

To clarify the relationship between Shh and the protein production of RANKL and IL-6, we added RU-SKI 43, an inhibitor of Shh signaling, to the culture of PDLFs with and without weights, and SDS-PAGE and Western blotting were performed. RU-SKI 43 did not inhibit the production of Shh but equally inhibited the production of RANKL and IL-6 in all conditions (Figure 10). The effects of RU-SKI 43 on the production of RANKL and IL-6 in the culture samples were quantitatively analyzed by ELISA. The inhibition by RU-SKI 43 on IL-6 production was significantly confirmed, but not for RANKL because the concentration of RANKL was too low to detect the protein in ELISA (Figure 11). RANKL was largely expressed on the cell surface and the production levels were low. The inhibition was not specific to responses to the production of RANKL and IL-6 elevated with the 1.0 g/cm^2^ and 4.0 g/cm^2^ weights.

## 4. Discussion

The various activities of cells in tissues are induced by light to heavy physiological pressures during orthodontic treatments. PDLFs are responsible for mechanical stress [28]. The tissue reactions are dependent on the amount of biomechanical load; whereas, moderate pressure is an integral part of periodontal tissue homeostasis, mechanical overload leads to cell damage associated with an increase in the PDLF death rate and dysregulation of bone remodeling [28,29,30]. According to various reports, the most favorable and efficient static compression magnitude is 2.0 g/cm^2^ (0.5 g/cm^2^ to 4.0 g/cm^2^) [31]. One point zero g/cm^2^ CF is within an optimal mechanical stress range for facilitating osteogenesis in osteoblasts by increasing type I collagen, bone sialoprotein, and bone morphogenetic protein expression levels; whereas, excessive mechanical stress (e.g., 4.0 g/cm^2^) attenuates these processes [32,33]. In this study, 1.0 g/cm^2^ and 4.0 g/cm^2^ weights were placed as an optimal orthodontic force and a strong orthodontic force on both PDLFs and HGFs with reference to previous research [23,24], respectively. The 1.0 g/cm^2^ and 4.0 g/cm^2^ weights did not lead to clear cell death and promoted suspended animation in PDLFs. In particular, the 4.0 g/cm^2^ weights resulted in greater quantities of detached cells than the 1.0 g/cm^2^ weights in both types of cells. We considered that weight would cause damage regardless of cell type. For the first 6 h after culture with weights in medium with 1% FBS, the effect of weight was less likely to be observed as a result of the influence of nutrient-rich medium with 10% FBS before the start of culture with weights.

Western blotting, a highly sensitive detection method using ECL Plus Western Blotting Substrate (Thermo Scientific) and X-ray film, was used to detect target substances. Production of soluble RANKL and IL-6 was clearly detected by Western blotting in the PDLF culture supernatant samples with weights. However, the production of RANKL was not measured due to the low production of soluble RANKL in the PDLF culture samples with weights. ELISA is a high-sensitivity immunoassay but has the limitation that samples need to fall within the range of the standard curve. Therefore, in these samples, Western blotting was more effective in measuring RANKL than ELISA. In the 6~24 h culture with weights, these cytokines were significantly expressed and produced compared to those of the control (glass plate only) group. In another report, under compression, the mRNA expression of IL-6 was upregulated in the first 24 h [23,24,34] and gradually decreased, with no significant differences from controls in the latter 24 h [35]. Therefore, the responses to sublethal stress depending on weight are common in PDLFs and involved in the production of RANKL and IL-6 in confluent PDLFs cultured for 6~24 h.

**Figure 12:**
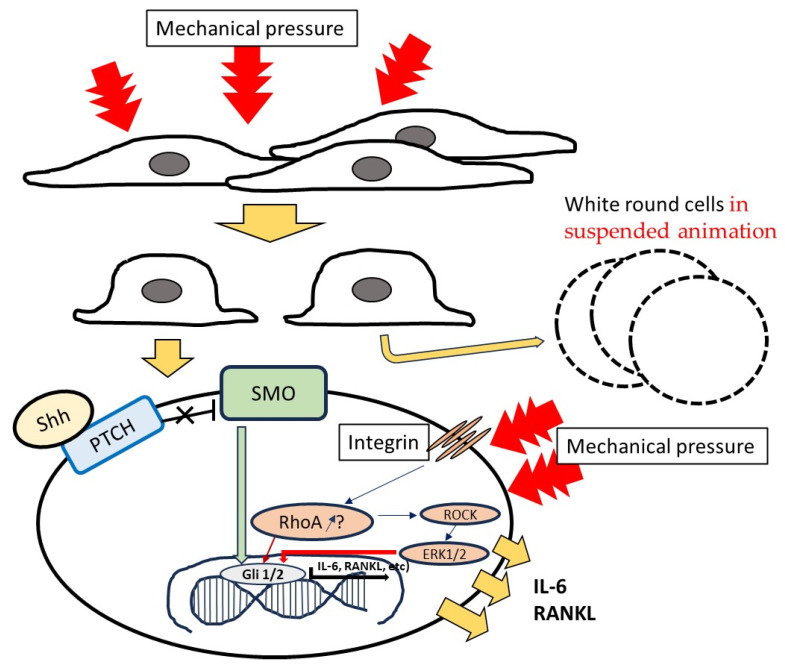
The summary schema of mechanical force- and Shh-dependent production of cytokines.

In periodontal tissue, PDLFs attach cement to neighboring alveolar bones, and fibrous connective tissue is involved in this process; 100s of collagen fibers, such as Sharpey’s and oxytalan fibers, are included [36]. PDLFs react sensitively to mechanical stimulation through interaction with the fiber-formed matrix [37,38,39]. In this study, HGFs, which were used as a control, reacted to mechanical stimulation, but the reactivities to weights for the production of RANKL and IL-6 were weaker than those of PDLFs. Therefore, the form, behavior, and function of PDLFs are strongly influenced by the structure of neighboring fibers and their location in PDLFs, including Sharpey’s or oxytalan fibers. The structure of PDL fibers is sensitively altered in response to a mechanical force, in which the crosslinks in collagen fibers and microfibrils are disrupted or condensed depending on compressive or tensile force application [36,40]. When PDLFs were exposed to compressive force on a tissue culture plate, some of the PDL collagen fibers showed a disrupted morphology. PDLF cultures under compressive mechanical force preferably induced morphological changes in cells and stimulated osteoclastic activity such as the production of RANKL and IL-6 rather than osteogenic differentiation. Hypofunctional periodontium is one of the major factors leading to dental root and bone resorption [8,9]. In this study, white round cells might have been induced by weight as a result of changes in fibrous morphology (Figure 12). White round cells are the shape of the cell when the PDLF is detached.

This shows the state in which fibroblasts return to their original form while floating when they detach and lose their ability to bind to the surface layer due to the force of physical weight. This may include cells with lost viability and cells having a high enough activity to produce cytokines for the induction of osteoclast. We considered that pressures such as those resulting from 1.0 g/cm^2^ and 4.0 g/cm^2^ weights would result in gene expression and produce cytokines for the induction of osteoclasts in sublethal responses, and, moreover, that the heavy force of a 4.0 g/cm^2^ weight might show more cell damage than the optimal force of a 1.0 g/cm^2^ weight. Then, PDLFs might be employed for their supportive role in osteoclastogenesis or odontoclastogenesis before induction in suspended animation because the white round cells that remained after 24 h of culture with 4.0 g/cm^2^ weights were stained by MTT and included active PDLF cells.

Hh signaling regulates several cellular processes that are critical to bone development [41]. The role of Hh signaling in regulating osteoclastogenesis is likely complicated in various tissues [42]. Shh is a key signaling molecule in the morphogenesis of various vertebrate organs, such as the limbs, the lower jaw, the heart tube, and the teeth [43,44,45,46]. Shh signaling has previously been shown to be involved in cell morphological changes and cell motility via Rho pathway-dependent myosin phosphorylation [47,48]. Myosin-dependent force generation not only increases Rac activity (the Rho family of GTPases) but also specifically increases lamellipodia along the sides and tail to decrease polarity [49]. Mechanical forces trigger multiple signaling pathways and result in the activation of the GTPase RhoA under the activity of integrins [50,51] (Figure 12). RhoA, a member of the Rho family, has been widely implicated in mechanosensitive signaling pathways [52,53]. The Shh signal transduction pathway downstream of SMO is mainly mediated by the activation of RhoA and is referred to as noncanonical type II signaling [54]. The RhoA protein plays a regulatory role in cytoskeletal components and contributes to osteoclast adhesion, podosome positioning, and differentiation [55]. Osteoclasts and odontoclasts are the most common mechanical sensors among these cells due to their structure and location in the bone and tooth matrix. Osteoclasts have been shown to sense damage-associated molecular patterns released by necrotic osteocytes [56]. Thus, mechanical loading RhoA-associated bone or root loss might be a result of crosstalk between PDLF and osteoclasts or odontoclasts.

The gene expression of Shh was not significantly associated with the gene expression of RANKL and IL-6 at time points after culture in PDLFs under weights. However, RU-SKI 43, which is an inhibitor of Shh signaling, inhibited the protein production of RANKL and IL-6, but not Shh itself, regardless of weight. For NKX6-1, a transcription factor that plays a critical role in pancreatic β cell function and proliferation, overexpression in LMS cells treated with RU-SKI43 resulted in cell growth inhibition [57]. The inhibition of Hh signaling in rats with arthritis using cyclopamine, another inhibitor of Shh, reduced the expression of TNFα, IL-1β, and IL-6 [41]. Shh indirectly increased osteoclast activity, resulting in increased bone resorption and decreased bone strength [11,12,13]. These reports support the indirect contribution of Shh to the effects of weight in the production of cytokines. The production of RANKL and IL-6 depending on weight might be mediated by RhoA in a mechanically sensitive signaling pathway; however, Shh is associated with cell growth and activity, ultimately guiding the production of cytokines in PDLFs.

Improper mechanical stress may induce morphological changes in PDLFs and the production of RANKL and IL-6 for bone and root resorption during orthodontic treatment. A heavy orthodontic force, such as the 4.0 g/cm^2^ weight, may cause more changes in morphology and root resorptions as a side effect, and induce unplanned tooth mobility and cause inflammation. However, Shh was not directly linked to signals for the weight-dependent production of cytokines. The study of Shh signal-independent systems, such as noncanonical type II signaling or the Shh signal transduction pathway downstream of SMO, is needed for the clarification of heavy weight-dependent cytokine production from PDLFs and side effects during orthodontic treatment. It is clear which signaling effects of noncanonical type II signaling or the Shh signal transduction pathway downstream of SMO are activated in response to weights, and targets may be identified to prevent side effects during orthodontic treatment.

## 5. Conclusions

PDLF cultures under compressive mechanical force preferably induced morphological changes in cells and stimulated osteoclastic activity. Shh did not directly link with signals for production of RANKL and IL-6 for root and bone resorptions by orthodontic force but was associated with cell activities that were finally guided by the production of cytokines in PDLFs.

## Figures and Tables

**Table 1:** Primers of target genes.

Gene	Forward (5′-3′)	Reverse (5′-3′)
Shh	GGACAGGCTGATGACTCAGA	GCCCTCGTAGTGCAGAGACT
RANKL	GCCTTTCAAGGAGCTGTGCAAAA	GAGCAAAAGGCTGAGCTTCAAGC
IL-6	AGACAGCCACTCACCTCTTCAG	TTCTGCCAGTGCCTCTTTGC
GAPDH	GTCAGTGGTGGACCTGACCT	TGCTGTAGCCAAATTCGTTG

## Data Availability

All data generated or analyzed during this study are included in this published article.

## Supplementary Materials

The following supporting information can be downloaded at: https://www.mdpi.com/article/10.3390/dj12040108/s1, Figure S1: Observation of white round cells and removed area. Figure S2: Observation of white round cells and removed area in different magnifications. Figure S3: Observation of cell viability.
