# Supplementary material for: Effects of Improper Mechanical Force on the Production of Sonic Hedgehog, RANKL, and IL-6 in Human Periodontal Ligament Cells In Vitro
Source: Dent J (Basel). 2024 Apr 15;12(4):108. doi: 10.3390/dj12040108 (PMC11049549; doi:10.3390/dj12040108)
Supplement: Supplementary file 1 [file dentistry-12-00108-s001.zip › dentistry-2823114-supplementary.pdf]

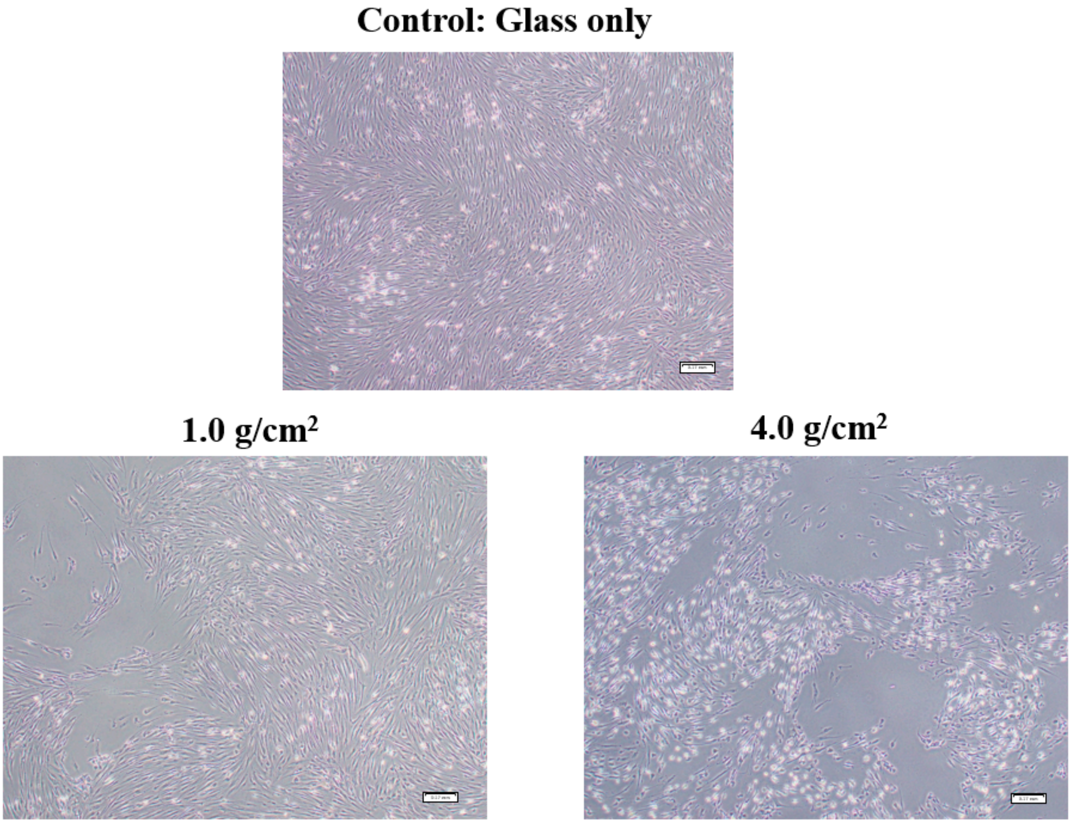

Figure S1. Observation of white round cells and removed area.

Observation of white round cells and removed area in compression force on the PDLF cells fibrosed layers. The optimal orthodontic force (1.0 g/cm^2) and the strong orthodontic force (4.0 g/cm^2) were placed on PDLF confluent cells in petri dish. Glass was only placed as a control. After 24 hours, PDLF cells were observed and taken pictures using a microscope. Scale bar indicate 5 mm. White round cells and removed area were clearly observed in the 4.0 g/cm^2 weight. Representative data from more than three independent experiments are presented in the pictures.

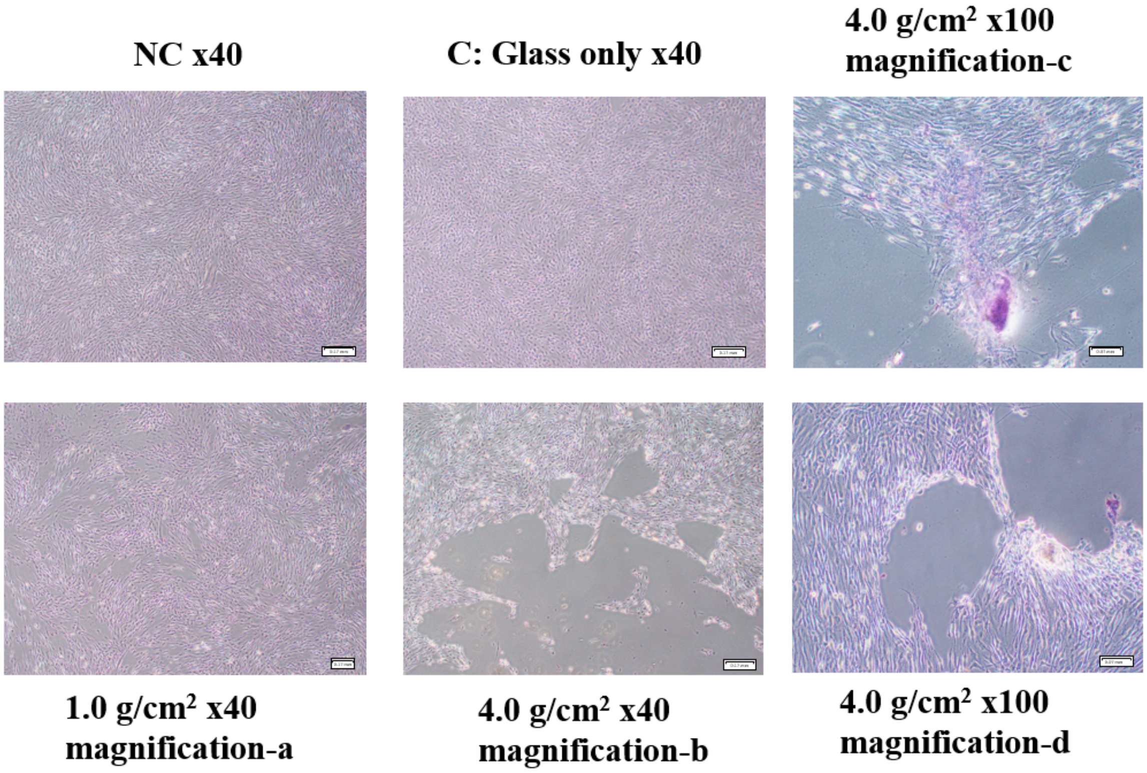

Figure S2. Observation of white round cells and removed area in different magnifications.

Observation of white round cells and removed area in compression force on the PDLF cells fibrosed layers in x40 and x100 magnifications. The optimal orthodontic force (1.0 g/cm^2) and the strong orthodontic force (4.0 g/cm^2) were placed on PDLF cells in petri dish. Glass was only placed as a control (C, x40). No glass and no weights were also performed as a negative control (NC, x40). After 24 hours, PDLF cells were observed and taken pictures using a microscope (x40 magnification -a and –b and x100 magnification -c and –d**)**. Scale bar indicate 5 mm and 2 mm in x40 and x100 magnifications. White round cells and removed area were clearly observed using x100 magnification -c and –d in PDLF cells placed with 4.0 g/cm^2 weight. Representative data from more than three independent experiments are presented in the pictures.

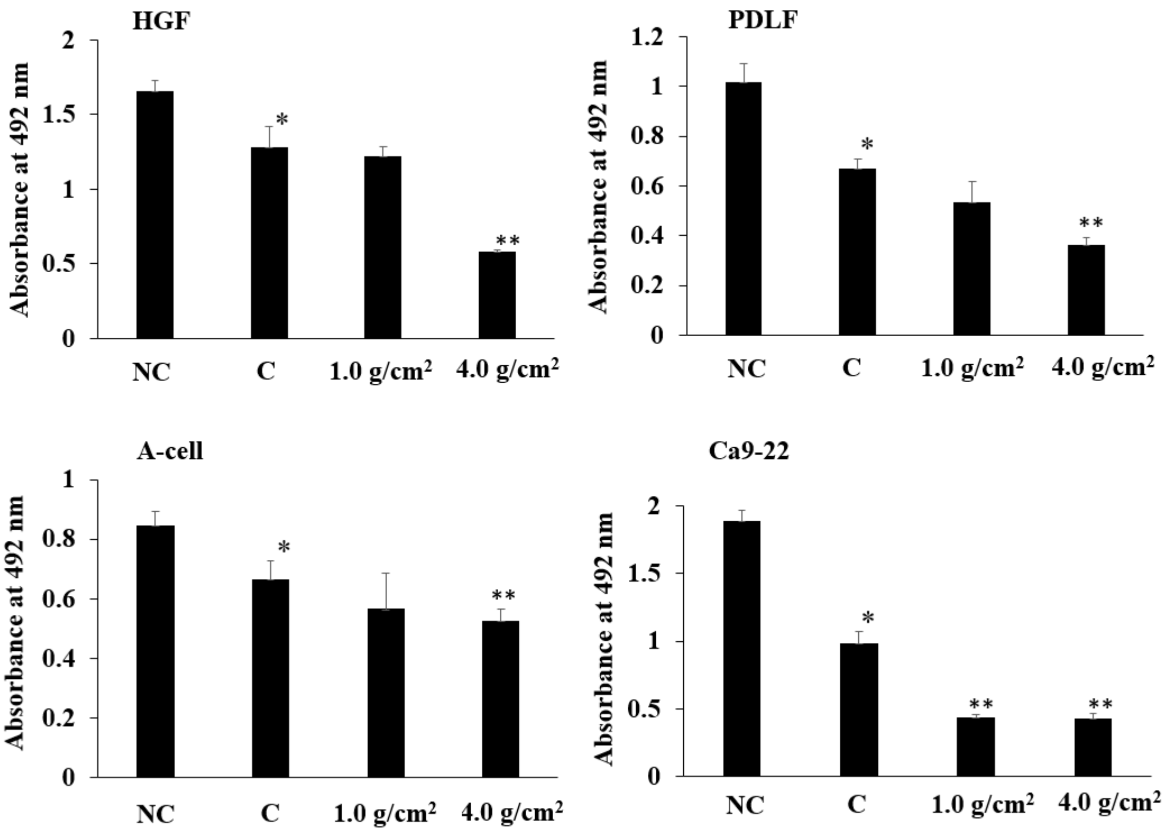

Figure S3. Observation of cell viability.

Observation of cell viability in the HGF, PDLF, A-cell and Ca9-22 cells fibrosed layer placed with weights. Cell viabilities were observed by MTT methods. The optimal orthodontic force (1.0 g/cm^2) and the strong orthodontic force (4.0 g/cm^2) were placed on various cells in petri dish. Glass was only placed as a control. No glass and no weights were also performed as a negative control (NC). After 24 hours, cells were treated by MTT and cell viability was measured by absorbance at 492 nm. The data indicate the mean ± SD of triplicate experiments. The independent experiments were performed two times, with similar results obtained in each experiment.
